# From gut to bone: deciphering the impact of gut microbiota on osteoporosis pathogenesis and management

**DOI:** 10.3389/fcimb.2024.1416739

**Published:** 2024-09-25

**Authors:** Linjie Hao, Yuzhu Yan, Guilin Huang, Hui Li

**Affiliations:** ^1^ Department of Joint Surgery, Honghui Hospital, Xi’an Jiaotong University, Xi’an, China; ^2^ Clinical Laboratory of Honghui Hospital, Xi’an Jiaotong University, Xi’an, China

**Keywords:** osteoporosis, gut microbiota, risk factors, short-chain fatty acids, fecal microbiota transplantation, therapeutic interventions

## Abstract

Osteoporosis (OP) is characterized by decreased bone mineral density (BMD) and increased fracture risk, poses a significant global health burden. Recent research has shed light on the bidirectional relationship between gut microbiota (GM) and bone health, presenting a novel avenue for understanding OP pathogenesis and developing targeted therapeutic interventions. This review provides a comprehensive overview of the GM-bone axis, exploring the impact of GM on OP development and management. We elucidate established risk factors and pathogenesis of OP, delve into the diversity and functional changes of GM in OP. Furthermore, we examine experimental evidence and clinical observations linking alterations in GM composition or function with variations in BMD and fracture risk. Mechanistic insights into microbial mediators of bone health, such as microbial metabolites and products, are discussed. Therapeutic implications, including GM-targeted interventions and dietary strategies, are also explored. Finally, we identify future research directions and challenges in translating these findings into clinical practice.

## Introduction

Osteoporosis (OP), a common skeletal disorder characterized by low bone mineral density (BMD) and microarchitectural deterioration of bone tissue, poses a significant public health concern globally (Pouresmaeili et al., 2018). It is associated with an increased risk of osteoporotic fractures (OPF, or fragility fractures, low-trauma fractures), resulting in substantial morbidity, mortality, and healthcare costs. The pathogenesis of OP involves an imbalance between bone resorption and formation processes, leading to compromised bone strength and increased susceptibility to fractures, particularly in the spine, hip, and wrist (Sozen et al., 2017). Factors contributing to OP include aging, hormonal changes (especially estrogen deficiency in postmenopausal women), genetic predisposition, nutritional deficiencies (e.g., calcium, vitamin D), sedentary lifestyle, and certain medications (e.g., glucocorticoids/GCS) (Bhattarai et al., 2020; Cheng et al., 2022a; Erdelyi et al., 2023; Smit et al., 2024). Other risk factors such as excessive alcohol consumption and tobacco use can also exacerbate the development of OP (Yang et al., 2021; Khiyali et al., 2024).

The gut microbiota (GM) refers to the complex community of microorganisms, including bacteria, viruses, fungi, and protozoa, that reside in the gastrointestinal tract. These microorganisms play crucial roles in multiple functions, including digestion, metabolism, and immune regulation, and are integral to host physiology and health (Thursby and Juge, 2017; Hou et al., 2022). The composition of GM is influenced by various factors, including diet, host genetics, age, medications, and environmental exposures. Dysbiosis is a term used to describe an imbalance in the GM. This imbalance can result from factors such as antibiotic use, poor diet, or illness and is associated with a range of health issues, including gastrointestinal disorders, metabolic conditions, and immune dysregulation. Alterations in GM composition and function, has been implicated in the pathogenesis of various diseases, including metabolic disorders like obesity and type 2 diabetes (Moreno-Indias et al., 2014), inflammatory conditions such as rheumatoid arthritis and inflammatory bowel disease (Li and Wang, 2021; Shan et al., 2022), gastrointestinal diseases like irritable bowel syndrome (Shaikh et al., 2023), and even neurological disorders like Parkinson’s disease and depression (Dinan and Cryan, 2017).

Recent research has highlighted the bidirectional interaction between GM and bone health, emphasizing the importance of deciphering the GM-bone axis in OP pathogenesis and management (Lyu et al., 2023). The GM influences bone metabolism through mechanisms such as nutrient absorption, immune modulation, and production of microbial metabolites. Understanding these interactions may reveal novel therapeutic targets for OP prevention and treatment. Moreover, dietary interventions and probiotics targeting GM composition hold promise for optimizing bone health and reducing the risk of OP or OPF. Exploring the GM-bone axis represents a paradigm shift in OP research, with implications for personalized approaches to disease management and improved clinical outcomes.

## Pathogenesis and risk factors of op

### Bone remodeling dynamics

Bone remodeling is a continuous physiological process responsible for maintaining skeletal integrity and adapting to mechanical stresses. This dynamic equilibrium between bone resorption and formation is tightly regulated by various cellular and molecular mechanisms, involving osteoclasts, osteoblasts, osteocytes, and bone matrix proteins (Bolamperti et al., 2022; Rowe et al., 2024). Osteoclasts, multinucleated cells originating from hematopoietic progenitors in the bone marrow, are specialized for bone resorption. They adhere to the bone surface and create an acidic microenvironment that facilitates the dissolution of the mineralized bone matrix through the secretion of hydrochloric acid and proteolytic enzymes such as cathepsin K, which results in the release of calcium and phosphate ions into the bloodstream (Bar-Shavit, 2007). Osteoblasts, on the other hand, are derived from mesenchymal stem cells (MSCs) and are responsible for the synthesis and deposition of new bone matrix, which subsequently undergoes mineralization to form mature bone tissue. This matrix is primarily composed of type I collagen and other non-collagenous proteins that provide the framework for mineral deposition (Florencio-Silva et al., 2015). Once osteoblasts become entrapped in the bone matrix, they differentiate into osteocytes, which play a crucial role in mechanotransduction and the regulation of both osteoclast and osteoblast activity through signaling pathways.

The equilibrium between bone resorption and formation is tightly regulated by a complex interplay of systemic and local factors. Systemic factors include hormones such as estrogen, which inhibits bone resorption by inducing osteoclast apoptosis and reducing the production of pro-resorptive cytokines; parathyroid hormone (PTH), which in low intermittent doses stimulates bone formation but in chronic elevation can increase bone resorption; and calcitonin, which directly inhibits osteoclast activity. Local factors involve cytokines like interleukin-6 (IL-6) and tumor necrosis factor-α (TNF-α), which promote osteoclast differentiation and activity, and growth factors such as insulin-like growth factor-1 (IGF-1) and transforming growth factor-β (TGF-β), which enhance osteoblast proliferation and function (Feng and McDonald, 2011; Singh et al., 2012). Disruption of this balance, characterized by increased bone resorption or decreased bone formation, results in bone loss and increased fracture risk, as observed in OP.

Mechanical loading also plays a pivotal role in bone remodeling by stimulating osteocytes to produce signaling molecules that regulate osteoblast and osteoclast activity (Robling and Turner, 2009; Du et al., 2020). Adequate mechanical loading is essential to maintain bone density and structure; conversely, reduced mechanical loading, as seen in sedentary lifestyles or prolonged bed rest, leads to bone loss.

Understanding the cellular and molecular mechanisms underlying bone remodeling provides insights into the pathogenesis of OP and highlights potential therapeutic targets for preventing and treating this condition. Efforts to modulate the activity of osteoclasts and osteoblasts, either through pharmacological agents or lifestyle interventions, are critical in maintaining bone health and reducing fracture risk in individuals with OP.

### Established risk factors for OP

**Figure 1:**
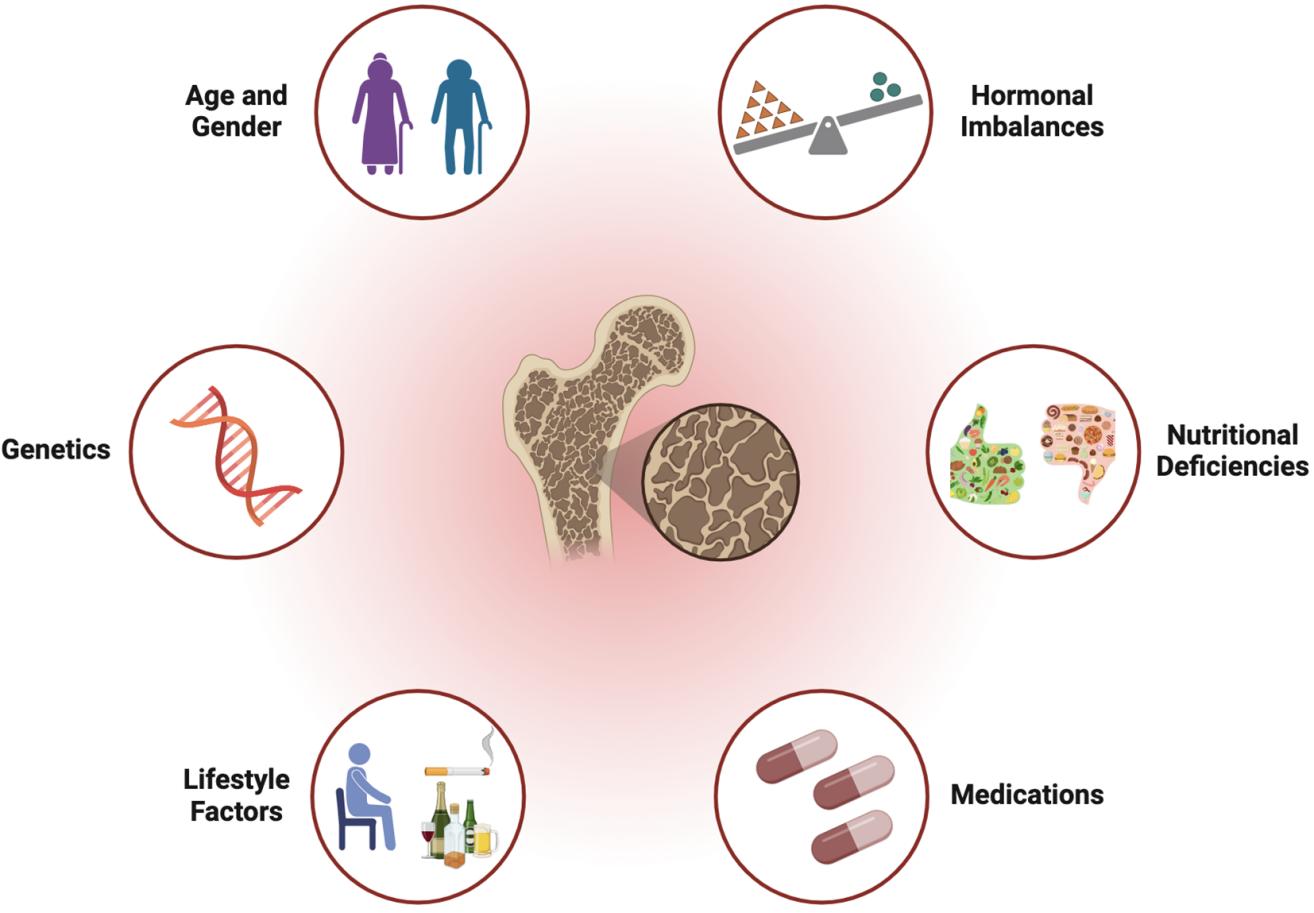
Established risk factors for osteoporosis.

As summarize in **Figure 1**, established risk factors for OP include aging, hormonal changes (especially estrogen deficiency in postmenopausal women), genetic predisposition, nutritional deficiencies (such as calcium and vitamin D), sedentary lifestyle, certain medications, excessive alcohol consumption, and tobacco use. These factors collectively contribute to the deterioration of BMD and increase the susceptibility to fractures and other skeletal complications.

#### Age

Aging is a major risk factor for OP, as bone mass typically peaks in early adulthood and gradually declines with age. Older adults are at increased risk of bone loss due to decreased osteoblast activity, hormonal changes, and cumulative exposure to other risk factors over time (Pignolo et al., 2021).

#### Gender

Women are at higher risk of OP compared to men, primarily due to the decline in estrogen levels associated with menopause. Estrogen plays a crucial role in maintaining BMD by suppressing osteoclast activity and promoting osteoblast function. Consequently, postmenopausal women experience accelerated bone loss and are more susceptible to OPF (Almeida et al., 2017; Charde et al., 2023).

#### Hormonal imbalances

Hormonal imbalances, including estrogen deficiency in women and androgen deficiency in men, contribute to OP development. Other endocrine disorders, such as hyperparathyroidism, hyperthyroidism, and adrenal insufficiency, can disrupt bone metabolism and increase fracture risk (Cheng et al., 2022a).

#### Genetics

Genetic factors play a significant role in determining BMD and fracture risk. Family history of OP or OPF, as well as genetic polymorphisms associated with bone turnover, mineralization, and skeletal structure, contribute to individual susceptibility to OP (Ralston, 2002; Makitie et al., 2019).

#### Nutritional deficiencies

Inadequate intake of calcium, vitamin D, and other essential nutrients essential for bone health increases the risk of OP. Calcium is required for bone mineralization, while vitamin D facilitates calcium absorption and bone metabolism regulation (Khazai et al., 2008).

#### Lifestyle factors

Sedentary lifestyle, smoking, excessive alcohol consumption, and low body mass index (BMI) are associated with increased OP risk. Physical inactivity and tobacco use adversely affect bone metabolism, while excessive alcohol intake interferes with calcium absorption and hormone levels (Padilla Colon et al., 2018; Niemela et al., 2022).

#### Medications

Certain medications, such as GCS, anticonvulsants, proton pump inhibitors (PPIs), and aromatase inhibitors (AIs), are associated with bone loss and increased fracture risk (Panday et al., 2014; Wang et al., 2023b). These medications may affect bone remodeling dynamics, calcium absorption, or hormone levels, leading to OP development.

## GM composition and function

### Diversity of gut microbiota

The GM is a complex ecosystem consisting of bacteria, archaea, fungi, viruses, and other microorganisms. Bacteria are the most abundant and extensively studied components of the GM, with thousands of different species identified to date. The composition of the GM varies significantly among individuals, influenced by factors such as age, diet, genetics, medications, and environmental exposures (Matijasic et al., 2020; Salazar et al., 2023).

Recent advancements in high-throughput sequencing technologies have enabled comprehensive characterization of GM composition at the taxonomic and functional levels (Wei et al., 2021a; Kwa et al., 2023). Key bacterial phyla inhabiting the human gut include Firmicutes, Bacteroidetes, Actinobacteria, Proteobacteria, Fusobacteria, and Verrucomicrobia (Rinninella et al., 2019). Within these phyla, numerous genera and species exhibit considerable diversity, contributing to the overall complexity of GM.

### Functions of GM in the gastrointestinal tract

#### Nutrient metabolism

GM contribute to the digestion and fermentation of dietary components, including complex carbohydrates, fiber, and resistant starches, producing metabolites such as short-chain fatty acids (SCFAs), amino acids, vitamins, and other bioactive compounds (Portincasa et al., 2022). SCFAs, particularly acetate, propionate, and butyrate, serve as energy sources for colonocytes, modulate immune responses, and influence host metabolism (Nogal et al., 2021).

#### Immune regulation

GM play a vital role in shaping host immune responses, maintaining immune homeostasis, and protecting against pathogens. Commensal bacteria interact with intestinal epithelial cells and immune cells, such as dendritic cells (DCs), macrophages, and T lymphocytes, influencing the development and function of the mucosal immune system (Zheng et al., 2020; Mazziotta et al., 2023). Dysbiosis, characterized by alterations in GM composition, has been associated with immune dysregulation and increased susceptibility to inflammatory and autoimmune diseases.

#### Barrier function

GM contribute to the maintenance of gut barrier integrity by promoting epithelial cell proliferation, enhancing mucin production, and modulating tight junction protein expression (Gierynska et al., 2022). By reinforcing the gut barrier, GM help prevent the translocation of harmful pathogens and microbial antigens from the intestinal lumen into systemic circulation, thereby reducing the risk of systemic inflammation and infection.

#### Metabolic regulation

GM influence host metabolism through various mechanisms, including energy harvest, regulation of lipid metabolism, bile acid metabolism, and modulation of glucose homeostasis (Ghazalpour et al., 2016; Martin et al., 2019). Alterations in GM composition and function have been implicated in the pathogenesis of metabolic disorders, such as obesity, insulin resistance, and type 2 diabetes mellitus (T2DM) (Crudele et al., 2023).

## Bidirectional interaction between GM and bone health

GM has emerged as a key regulator of bone metabolism, exerting both direct and indirect effects on bone health. Understanding the bidirectional interaction between GM and bone is essential for elucidating the mechanisms underlying OP pathogenesis and developing novel therapeutic strategies (Wang et al., 2022f; Lyu et al., 2023).

### Influence of GM on bone metabolism

Evidences from preclinical and clinical studies indicate that GM composition influences bone metabolism and contributes to variations in BMD and bone strength. Experimental studies in germ-free (GF) animal models, devoid of GM, have demonstrated altered bone phenotypes characterized by decreased BMD, impaired bone microarchitecture, and compromised bone strength compared to conventionally raised animals. Reconstitution of GF animals with specific microbes or microbial metabolites has been shown to partially restore BMD and integrity, highlighting the role of GM in regulating bone metabolism. Furthermore, alterations in GM composition, induced through dietary interventions, probiotics, antibiotics, or fecal microbiota transplantation (FMT), have been associated with changes in bone turnover markers (BTMs), calcium absorption, and skeletal phenotypes in animal models. Clinical and animal studies have also reported associations between GM dysbiosis and OP prevalence, low BMD, and fracture risk in humans (refer to **Tables 1**, **2** below). Collectively, these findings support the notion that GM play a significant role in modulating bone metabolism and influencing skeletal health.

**Table 1:** Summary of animal studies investigating or linking the effects of GM on bone metabolism.

Animal models	Main findings	Refs
Aging mice	Dietary intervention with diallyl trisulfide alleviates age-related bone loss by improving bone microstructure, promoting collagen synthesis, and upregulating osteogenic gene expression, potentially mediated by alterations in GM composition and serum metabolism.	(Zhang et al., 2023a)
Aging mice	Fructus Ligustri Lucidi mitigated OP and improved cognitive function by modulating GM diversity, antioxidant activity, and levels of TMAO and Sirtuin 6.	(Li et al., 2019)
Aging mice	*Lactobacillus plantarum* TWK10 (TWK10) improved muscle strength, prevented aging-related loss of muscle strength, attenuated decline in learning and memory abilities, preserved bone mass, altered GM composition, increased SCFAs-producing bacteria, and reversed aging-associated accumulation of pathogenic bacterial taxa in mice.	(Lee et al., 2021)
Aging mice	Gut microbiota transplantation (GMT) from old mice reduces lean mass percentage but does not significantly affect bone mass in young recipient mice.	(Lawenius et al., 2023)
Aging mice; OVX mice	D-mannose attenuated bone loss induced by senility and estrogen deficiency in mice, mediated by increased Treg and GM-dependent anti-inflammatory effects.	(Liu et al., 2020a)
Aging rats	The study investigated the association between GM and senile OP, revealing reduced alpha diversity, altered F/B ratio, and enrichment of Helicobacter related to OP.	(Ma et al., 2020a)
Aging rats	Senile OP rats exhibited altered GM composition, characterized by decreased diversity, increased abundance of certain species, along with enrichment of metabolic pathways.	(Wang et al., 2022c)
Aging rats	FMT from young rats alleviated bone loss in aged rats with senile OP by improving GM composition and intestinal barrier function.	(Ma et al., 2021)
Alcohol-induced OP rats	Long-term alcohol consumption induced OP, with more severe effects observed in older rats compared to youngers. Alcohol consumption also altered GM composition, exacerbating OP through activation of T lymphocytes and cytokine production, particularly in older rats with lower GM diversity and regulatory capacity.	(Cheng et al., 2021)
DEX-induced OP rats	Epigallocatechin gallate prevented bone loss induced by dexamethasone (DEX), preserved BMD and microstructure, increased GM diversity.	(Han et al., 2023)
DEX-induced OP rats	*Astragalus* polysaccharides were found to restore BMD, repair bone microarchitecture, decrease ACP5 and pro-inflammatory cytokines, and modulate GM composition.	(Liu et al., 2020b)
DEX-induced OP rats	Fermentation of Astragalus polysaccharides with Lactobacillus acidophilus improved calcium absorption and OP more effectively than the unfermented mixed solution, through alterations in GM composition and increased levels of active metabolites.	(Zhou et al., 2023)
DIO rats	The study identified alterations in GM abundance and fecal metabolites in disuse-induced osteoporosis (DIO) rats.	(Qiao et al., 2022)
EIO rats	Chronic ethanol led to OP (ethanol-induced osteoporosis, EIO) and intestinal dysbiosis, with increased serotonin positively correlating with GM and metabolites changes.	(Liu et al., 2022)
FMT rats	Transfer of GM from senile OP rats to young rats induced OP, increased BTMs, decreased bone volume, altered GM composition, and impaired intestinal barrier integrity.	(Wang et al., 2022b)
GF mice	GM modulates inflammatory responses induced by sex steroid deficiency, leading to trabecular bone loss, and probiotic treatments effectively reduce gut permeability, intestinal and bone marrow inflammation, and protect against bone loss.	(Li et al., 2016)
GF mice	GM regulates bone mass by modulating the immune status in bone marrow, affecting osteoclast-mediated bone resorption.	(Sjogren et al., 2012)
GF mice	Long-term antibiotic feeding alters GM composition, reduces BMD and leads to changes in trabecular microstructure, accompanied by decrease in estrogen levels.	(Guo et al., 2022a)
GF mice	Despite successful colonization with GM of either mouse or human origin, microbial colonization did not significantly alter bone mass or related parameters in GF mice.	(Quach et al., 2018)
GIOP mice	The study demonstrates that glucocorticoid-induced osteoporosis (GIOP) is mediated by alterations in GM composition and intestinal barrier function, with microbiota depletion or probiotic treatment preventing trabecular bone loss and restoring Wnt10b expression.	(Schepper et al., 2020)
GIOP mice	Tuna bone powder demonstrates efficacy in alleviating GIOP mice by modulating signaling pathways, suppressing pro-inflammatory cytokines, repairing the intestinal barrier, and enhancing the abundance of anti-inflammatory gut bacteria and SCFAs.	(Li et al., 2020b)
GIOP mice	Korean Red Ginseng extract prevented glucocorticoid-induced bone loss by modulating GM composition, gut barrier function and immune cell populations.	(Chargo et al., 2024)
GIOP rats	*Lactobacillus plantarum* improved bone microstructure, increased GM diversity, altered GM composition, and modulated metabolites related to bone metabolism.	(Li et al., 2023)
GIOP rats	Oral administration of Lactobacillus plantarum LP45 prevented bone defects in GIOP rats, improving bone histomorphometry, BMC, BMD, and femoral biomechanics. Additionally, LP45 restored the imbalance in BTMs and the RANKL/OPG signaling pathway.	(Jiang et al., 2023)
HFD mice	The study revealed that high-fat diet (HFD) induced bone loss in mice, accompanied by expansion of bone marrow adipose tissue and inhibition of bone formation. Analysis showed significant alterations in GM composition and serum metabolites associated with HFD-induced bone loss.	(Lu et al., 2021a)
HFD mice	Long-term HFD induced bone loss, associated with GM dysbiosis, increased gut permeability, and systemic inflammation; treatment with fructooligosaccharides (FOS) and/or galactooligosaccharides (GOS) alleviated bone loss by restoring GM diversity, reducing gut permeability, and decreasing inflammation.	(Zhang et al., 2021)
Hindlimb unloaded mice	Asperosaponin VI improved bone microarchitecture in by reversing bone loss indicators and regulating specific GM, particularly Clostridium, and its metabolites.	(Niu et al., 2023)
Hindlimb unloaded rats	Cordymin treatment in increased bone mechanical strength and bone volume while regulating GM composition, improving BMD and reducing trabecular separation.	(Qi et al., 2024)
Mice	Eclipta prostrata could prevent OP by modulating GM, inhibiting osteoclasts, increasing osteoblasts, and regulating bone absorption and formation.	(Zhao et al., 2019)
Mice	Oral administration of Lactobacillus rhamnosus GG attenuated tenofovir disoproxil fumarate-induced bone loss by promoting trabecular bone microarchitecture, cortical bone volume, intestinal barrier integrity, Treg expansion, and downregulating osteoclastogenesis-related cytokines, through GM modulation and altered metabolite composition.	(Liu et al., 2019)
Mice	Antibiotic-induced dysbiosis in mice led to lower BMD and increased serum levels of RANKL and Ang II. Probiotic treatment promoted fracture healing in these mice, potentially by inhibiting the RAS/RANKL/RANK pathway.	(Guo et al., 2022b)
Mice	Exercise prevented HFD-induced bone pathology, improving trabecular bone volume and reducing marrow adiposity, altered the GM composition by reducing the F/B ratio.	(McCabe et al., 2019)
OVX mice	FMT mitigated ovariectomy-induced bone loss by modulating GM composition, enhancing intestinal barrier function, and reducing pro-osteoclastogenic cytokine release.	(Zhang et al., 2022b)
OVX mice	GM composition, particularly the Firmicutes/Bacteroidetes (F/B) ratio, is closely linked to OP development. Furthermore, probiotic supplementation with *Lactobacillus salivarius* LI01 from the Firmicutes phylum was effective in preventing OP by modulating glutathione synthesis and reducing reactive oxygen species.	(Yuan et al., 2022)
OVX mice	OVX mice fed Bacteroides vulgatus exhibited increased bone resorption and poorer bone microstructure.	(Lin et al., 2023)
OVX mice	Warmth exposure at 34°C protects against ovariectomy-induced bone loss by increasing trabecular bone volume, connectivity density, and thickness, with warmth and warm microbiota transplantation reversing transcriptomics changes, enhancing periosteal bone formation, and promoting bacterial polyamine biosynthesis.	(Chevalier et al., 2020)
OVX mice	The study reveals that gold nanospheres prevent ovariectomy-induced OP by modulating GM diversity and composition, reducing trimethylamine-N-oxide (TMAO)-related metabolites, and inhibiting pro-osteoclastogenic and proinflammatory cytokine release.	(Chen et al., 2023c)
OVX mice	Dietary isoquercetin improves OP by modulating GM, improving gut barrier function, suppressing inflammatory cytokines, promoting osteoblast proliferation/differentiation.	(Wu et al., 2023)
OVX mice	Velvet antler extract administration in OVX mice leads to improved bone-related biochemical markers in serum, enhanced bone microstructure, and modulation of GM.	(Pan et al., 2023)
OVX mice	GM imbalance exacerbates bone loss, depletion of GM improves bone mass and strength, with targeting the G-protein-coupled bile acid receptor (TGR5).	(Guan et al., 2023)
OVX mice	Treatment with Prevotella histicola prevents estrogen deficiency-induced bone loss by modulating gut permeability and inhibiting osteoclast activity.	(Wang et al., 2021)
OVX mice	Oral administration of kefir-fermented peptides prevents PMOP, accompanied by modulation of GM structure, including restoration of certain bacterial genera to normal levels.	(Tu et al., 2020)
OVX mice	Compound deer bone extract (CDBE) improved serum bone-related biochemical indicators, trabecular microstructure, and intestinal flora in OVX mice.	(Xue et al., 2021)
OVX mice	(R)-ketamine administration ameliorated the reduction in cortical and total BMD, through its anti-inflammatory effects mediated by changes in the GM composition.	(Wan et al., 2022)
OVX mice	Bovine raw milk-derived extracellular vesicles inhibited osteoclast differentiation, improved microarchitecture, restored osteoporotic biomarkers, enhanced intestinal permeability, reduced endotoxin levels, modulated GM composition, increased SCFAs, and decreased pro-inflammatory cytokines and osteoclast differentiation-related factors.	(Hao et al., 2024)
OVX mice	Heat-killed Lacticaseibacillus paracasei GMNL-653 exhibited anti-inflammatory effects, restored GM dysbiosis, maintained intestinal barrier integrity, reduced inflammatory markers, modulated bone-related gene expression, influenced host metabolic pathways, and potentially involved specific genes in antiosteoporotic activity.	(Jhong et al., 2022)
OVX mice	Supplementation of acid-hydrolysed high amylose corn starch (AH-HAS) in diets increased Bifidobacterium spp. abundance, upregulated IL-10 expression in the colon, downregulated receptor activator of NF-κB ligand and IL-7 receptor genes in bone marrow, and attenuated ovariectomy-induced bone loss.	(Tousen et al., 2019)
OVX mice	*Lactobacillus plantarum* NK3 and Bifidobacterium longum NK49 administration alleviated Gardnerella vaginalis-induced vaginosis and ovariectomy-induced OP in female mice by suppressing NF-κB-linked TNF-α expression through the regulation of GM.	(Kim et al., 2019)
OVX mice	Estrogen deficiency-induced disruption of GM composition leads to intestinal barrier dysfunction and gut leakage, contributing to osteoclastogenesis. Icariside I (GH01) treatment restores GM composition, intestinal barrier function, and host immune status, effectively ameliorating bone loss and OP by targeting the gut-bone signaling axis.	(Chen et al., 2023a)
OVX mice	Regular and quantitative perfusion of Prevotella histicola (Ph) mitigated bone loss in OVX-induced OP mice by suppressing osteoclastogenesis, promoting osteogenesis, reducing pro-inflammatory cytokine release, and improving GM composition and diversity.	(Zhang et al., 2023c)
OVX mice	Ovariectomy-induced OP was associated with lower BMD and altered serum bone marker levels, along with changes in GM composition and increased intestinal inflammation. However, remodeling the GM through antibiotic treatment and FMT did not significantly impact OP outcomes, suggesting a minor role of GM in this condition.	(Kosaka et al., 2021)
OVX mice	Orally administered lactulose prevented ovariectomy-induced bone loss by inhibiting osteoclastogenesis, increasing intestinal tight junction proteins, modulating pro- and anti-inflammatory cytokines, preserving Treg cells, altering GM composition, and increasing SCFAs.	(Chen et al., 2020b)
OVX mice	Cinnamic acid demonstrated enhanced osteoblast differentiation, improved BMD, increased GM diversity, and restored GM composition changes induced by ovariectomy.	(Hong et al., 2022)
OVX mice	Treatment with Bacteroides vulgatus ATCC 8482 in ovariectomized mice ameliorated bone loss and microstructure destruction in the lumbar vertebra by reducing GM dysbiosis, down-regulating colonic inflammation pathways, decreasing serum TNF-α levels, and inducing expression of BTMs ALP and Runx2.	(Yuan and Shen, 2021)
OVX mice	Decreased abundance of Clostridium sporogenes (C. spor.) and its metabolite, indole propionic acid (IPA), was observed in OVX mice. IPA suppressed osteoclast differentiation by stabilizing pregnane X receptor (PXR) and enhancing its binding with P65, ultimately protecting against estrogen deficiency-induced bone loss. Oral administration of C. spor.-encapsulated silk fibroin hydrogel or IPA mitigated OVX-induced bone loss.	(Peng et al., 2024)
OVX mice	*Bifidobacterium* treatment improved BMD, bone volume, and trabecular number by suppressing inflammation, osteoclast generation, and enhancing mucosal barrier protection.	(Zhang et al., 2024)
OVX mice	Nodakenin treatment improved bone microstructure, BTMs, and intestinal mucosal integrity in OVX mice by modulating the GM composition and metabolites.	(Liu et al., 2024)
OVX mice	*Lactobacillus brevis* AR281 significantly improved bone microarchitecture and biomechanical strength in OVX mice by attenuating bone resorption, decreasing the RANKL/OPG ratio and pro-inflammatory mediators, modulating GM, and suppressing osteoclastogenesis through the TRAF6/NF-κB/NFATc1 pathway.	(Yu et al., 2022)
OVX mice	Agastache rugosa demonstrated therapeutic effects on OP by promoting osteoblast differentiation, suppressing bone loss, elevating osteogenic markers, reversing GM changes.	(Hong et al., 2021)
OVX mice	Rothia alleviated bone loss by repairing intestinal mucosal barrier injury, optimizing intestinal permeability, reducing intestinal inflammation, and regulating GM imbalance	(Li et al., 2024)
OVX mice	GM depletion in mice protected against bone loss and cartilage degradation in OP by modulating the composition of the GM, resulting in increased BMD, bone volume fraction, trabecular number, and decreased levels of inflammatory markers.	(Yin et al., 2024)
OVX mice	*Lactobacillus reuteri* protected OVX mice from bone loss by suppressing osteoclast bone resorption markers and activators, reducing osteoclastogenesis, and modifying GM.	(Britton et al., 2014)
OVX mice	BX, derived from Psoralea corylifolia L., alleviated OP by improving bone parameters and bone formation markers. BX also modulated GM and restored metabolic disorders.	(Wei et al., 2024)
OVX rats	*Lactobacillus rhamnosus* GG (LGG) treatment alleviated estrogen deficiency-induced OP by promoting osteogenesis, modulating Th17/Treg balance, improving intestinal barrier function, and regulating the GM composition.	(Guo et al., 2023)
OVX rats	Exogenous overexpression of neuropeptide Y exacerbated bone loss and colonic inflammation, impaired intestinal barrier integrity and altered GM composition.	(Chen et al., 2023d)
OVX rats	Qing’e Pills administration in OVX rats increased BMD, altered GM composition, inhibited inflammatory factors TNF-α and IL-6, and increased levels of SCFAs.	(Xie et al., 2022)
OVX rats	Moringa oleifera leaf (MLP) significantly increased BMD, improved bone metabolism-related indicators and bone microstructure, modulated GM composition, and activated ERK and VAV3 protein expression while decreasing p-ERK and JNK protein expression.	(Hu et al., 2023)
OVX rats	Icariin (ICA) administration in OVX rats improved bone microarchitecture and correlated with changes in GM composition and fecal metabolites.	(Wang et al., 2022e)
OVX rats	Puerarin treatment in OVX rats improved bone density and integrity by modulating GM composition, increasing SCFAs, and repairing intestinal mucosal integrity.	(Li et al., 2020a)
OVX rats	Quinoa improved OP-related biochemical parameters, bone density, and trabecular structure, by repairing intestinal barrier function, regulating GM composition, and influencing various metabolic pathways.	(Dou et al., 2024)
OVX rats	Chondroitin sulfate calcium complex (CSCa) improved BMD, femur microstructure, and serum BTMs. Besides, it altered GM composition and fecal metabolites.	(Shen et al., 2021)
OVX rats	Ovariectomy led to significant changes in GM composition and function, with increased diversity and taxonomic differences observed.	(Ma et al., 2020b)
OVX rats	Calcium supplementation combined with inulin positively influences GM composition and function, leading to improved BMD, bone mineral content (BMC), femur mechanical strength, and decreased serum bone markers.	(He et al., 2022)
OVX rats	Konjac oligosaccharides (KOS) effectively mitigated bone loss by promoting gut barrier repair, reducing pro-inflammatory cytokines, promoting the growth of beneficial gut bacteria like Bifidobacterium longum, and restoring Treg/Th17 balance in bone marrow.	(Ai et al., 2024)
OVX rats	Arecanut seed polyphenol (ACP) improved trabecular microstructure, associated with increased expression of lysozyme and maintenance of Paneth cells, leading to modulation of GM composition and improvement of OP by controlling inflammatory reactions.	(Mei et al., 2021)
OVX rats	Jiangu granule restored GM composition, increased SCFAs, promoted Treg cell proliferation, and modulated cytokine levels, ultimately preventing bone loss and enhancing bone strength through the “GM-SCFAs-Treg/Th17″ axis.	(Sun et al., 2022)
OVX rats	Diosgenin improved bone microstructure and prevented weight gain, also modulating the composition and function of GM.	(Song et al., 2023)
OVX rats	GM alterations at the species level, including increased abundance of Helicobacter rodentium, Lachnospiraceae bacterium 10 1, and Lachnospiraceae bacterium A4, were observed in OVX rats, along with changes in functional metabolism pathways.	(Wang et al., 2022d)
OVX rats	Parathyroid hormone improved bone parameters and altered GM composition and function, increasing abundance of probiotic bacteria and reducing pathogenic bacteria.	(Zhou et al., 2022)
OVX rats	Probiotic attenuated inflammatory alveolar bone loss by modulating GM, improving gut barrier function, suppressing oestrogen deprivation-induced inflammatory.	(Jia et al., 2021)
OVX rats	Berberine reduced alveolar bone loss and improved bone metabolism by increasing butyrate-producing GM, enhancing intestinal barrier integrity, reducing intestinal permeability, and attenuating IL-17A-related immune responses.	(Jia et al., 2019)
OVX rats	Soy-whey dual-protein improved BMD, microstructure, and biomechanics, reduced serum OCN and PTH levels, decreased bone marrow adipocytes while increasing osteoblasts, and regulated key regulatory factors like osteoprotegerin. DP altered fecal metabolites and GM, influencing fat metabolism-related molecules and bacterial taxa.	(Zhang et al., 2022a)
OVX rats	Supplementation of white LED exposure with infrared light positively affected bone metabolism by altering GM.	(Lu et al., 2021b)
OVX rats	Erythrina cortex extract demonstrated bone protective effects by improving BMD and microarchitecture, modulating GM composition and increasing serum levels of SCFAs.	(Xiao et al., 2021)
OVX rats	*Bifidobacterium longum* supplementation increased BMD, BMC, and bone formation parameters while decreasing bone resorption, ultimately alleviating bone loss.	(Parvaneh et al., 2015)
PMOP mice	Changes in the GM and metabolites in feces and serum were identified as crucial factors in the occurrence and development of PMOP.	(Wen et al., 2020)
SAMP6 mice	Dietary supplementation with fructooligosaccharide and glucomannan modified GM, increased calcium content in femoral bones, and reduced bone resorption and systemic inflammation in senescence-accelerated mouse prone 6 (SAMP6) mice.	(Tanabe et al., 2019)
SAMP6 mice	Eucommia ulmoides leaf extract increased GM diversity and F/B ratio, elevated SCFAs, and ameliorated OP.	(Zhao et al., 2020)
SCD mice	Antibiotic treatment in sickle cell disease (SCD) mice rescued bone loss by improving intestinal barrier function, reducing inflammation, and enhancing osteoblast function, suggesting a link between GM dysbiosis and bone loss in SCD.	(Tavakoli and Xiao, 2019)
TLR5KO mice	Chronic antibiotic treatment disrupted the GM, leading to impaired bone tissue material properties and reduced bone strength, independent of changes in bone geometry.	(Guss et al., 2017)
TLR9-/- mice	TLR9 deletion leads to low bone mass and chronic inflammation characterized by CD4+ T cell expansion and elevated inflammatory cytokines, promoting osteoclastogenesis and bone loss. Dysbiosis in the GM contributes to systemic inflammation and bone loss in TLR9-/- mice, while increased myelopoiesis in the bone marrow exacerbates inflammation-induced osteoclastogenesis and bone loss.	(Ding et al., 2022)
UC mice	Bifidobacterium lactis BL-99 alleviates dextran sodium sulfate-induced ulcerative colitis (UC), reduces inflammation, preserves intestinal barrier, and prevents bone loss.	(Lan et al., 2022)

**Table 2:** Summary of clinical studies examining GM alterations or interventions among patients with low BMD.

Participants or Patients	Summaries	Refs
Elderly Chinese Han individuals	Common variants in the R-spondin/Wnt signaling genes, particularly rs10920362 in LGR6 and rs11178860 in LGR5, are associated with OP risk. Alterations in GM composition, particularly Actinobacteria, Bifidobacteriaceae, and Bifidobacterium, are linked to these genetic variants and OP risk.	(Di et al., 2021)
Elderly individuals	Elderly patients with fragility hip fractures had specific alterations in their gut microbiota composition compared to controls.	(Rosello-Anon et al., 2023)
OP and ON patients	Microbial taxa, including Firmicutes, Bacteroidetes, Gemmatimonadetes, Chloroflexi, Blautia, Parabacteroides, and Ruminococcaceae, differ between OP and controls.	(Wang et al., 2017)
OP patients	The study found that differences in GM composition, particularly lower abundance of Bifidobacterium, are associated with reduced absorption of cholecalciferol and lower circulating concentrations of 25(OH)D3 in patients with severe OP compared to primary OP.	(Cheng et al., 2022b)
OP patients	*Bacillus acidophilus* plays a role in OP by modulating GM diversity and influencing the proliferation, differentiation, and maturity of osteoblasts and osteoclasts.	(Chen et al., 2020a)
OP patients	Yigu decoction positively influence the GM structure and function in OP patients, led to improvements in BMD and induced changes in GM composition and metabolites.	(Zhang et al., 2023b)
OP patients	The study establishes a causal link GM and OP using two-sample Mendelian randomization analysis, identifying specific GM taxa associated with the risk of OP.	(Zeng et al., 2024)
OP patients	The study found that OP individuals exhibited alterations in GM composition compared to controls, with specific taxa associated with reduced BMD.	(Wei et al., 2021b)
OP patients	Elevated levels of GM metabolite TMAO are correlated with decreased BMD in OP patients. TMAO regulates BMSCs function by activating the NF-κB signaling pathway, leading to enhanced adipogenesis, reduced osteogenesis, increased reactive oxygen species (ROS) release, and elevated pro-inflammatory cytokine production.	(Lin et al., 2020)
OP patients	OP is associated with alterations in GM composition, particularly enrichment of Dialister and Faecalibacterium genera.	(Xu et al., 2020)
OP patients	The study identifies the genus Lachnospira as a potential biomarker for OP.	(Ul-Haq et al., 2022)
PMOP patients	GM composition differs in PMOP patients compared to controls, with microbial abundances correlating more strongly with total hip than lumbar spine BMD/T-score. Using feature selection, Fusobacteria and Lactobacillaceae were identified as significant microbial markers for disease classification between PMOP and control groups.	(Huang et al., 2023)
PMOP patients	The study demonstrates that reduced GM diversity and elevated levels of fecal glycolithocholic acid (GLCA) correlate with PMOP severity. GLCA supplementation alleviates OP by increasing circulating Tregs, which in turn promote osteogenic differentiation of BMSCs.	(Cai et al., 2024)
Postmenopausal women	The study identified *Bacteroides vulgatus* as a species negatively associated with BMD in peri-/post-menopausal Chinese women, with validation in US white populations.	(Lin et al., 2023)
Postmenopausal women	PMOP exhibited significant alterations in GM composition, fecal metabolites, and associated signaling pathways compared to non-OP postmenopausal women.	(Wang et al., 2023a)
Postmenopausal women	Postmenopausal women with OP exhibit distinct variations in GM and vaginal microbiota (VM) compared to those with osteopenia (ON) or normal BMD.	(Yang et al., 2022)
postmenopausal women and men aged over 50	The study elucidates the GM composition and gene functional profile in older individuals with normal and low BMD, particularly highlighting differences in composition and gene abundance between genders. Additionally, the study underscores the importance of addressing vitamin D deficiency or insufficiency in the Chinese population.	(Wang et al., 2022f)
Postmenopausal women	The study demonstrates a significant association between elevated GM metabolite TMAO and increased risk of hip fracture in postmenopausal women.	(Liu et al., 2020c)
Two large cohorts	The study investigates the association between GM and skeletal health using human cohorts, revealing significant associations between GM abundances and bone measures.	(Okoro et al., 2023)
T2DM patients	The study reveals association between elevated TMAO and reduced BMD, as well as increased risk of OP and OPF in patients with T2DM.	(Yuan et al., 2024)

### Mechanisms of GM-bone axis

The mechanisms of the GM-bone axis involve multiple pathways. Firstly, GM contribute to nutrient metabolism and absorption, particularly calcium and vitamin D, essential for bone mineralization and remodeling (Wang et al., 2022a). Secondly, GM interact with the host immune system, influencing local and systemic immune responses, with dysbiosis potentially leading to chronic inflammation and bone loss (D’Amelio and Sassi, 2018). Thirdly, GM produce various metabolites such as SCFAs and bile acids, which act as signaling molecules affecting host metabolism and immune function, thereby influencing bone remodeling processes (Yoon et al., 2023). Additionally, GM influence hormonal regulation pathways involved in bone metabolism, including estrogen, vitamin D, and PTH signaling, with dysbiosis potentially disrupting hormone levels and receptor activation, leading to imbalances in bone homeostasis and increase fracture risk (Iwobi and Sparks, 2023). Lastly, GM produce microbial-derived factors like lipopolysaccharides (LPS) and extracellular vesicles, which interact with host cells and modulate inflammatory and signaling pathways relevant to bone remodeling (Chen et al., 2023b).

## Experimental evidence and clinical observations

### Findings from animal studies

Animal studies have provided valuable insights into the relationship between GM and bone health. GF animals, devoid of gut microbiota, exhibit altered bone phenotypes characterized by reduced BMD and compromised bone microarchitecture compared to conventionally raised animals. Experimental manipulation of GM composition through dietary interventions, probiotics, antibiotics, or FMT has further elucidated the impact of GM on bone metabolism. Reconstitution of GF animals with specific microbial strains or microbial metabolites has been shown to attenuate bone loss and improve skeletal phenotypes, highlighting the therapeutic potential of modulating GM for bone health. Mechanistic studies in animal models have revealed potential pathways through which GM influence bone metabolism, including nutrient absorption, immune modulation, and production of microbial metabolites. SCFAs, bile acids, and secondary bile acids derived from gut microbial fermentation have been implicated as key mediators of the GM-bone axis, affecting osteoclast and osteoblast activity, bone remodeling dynamics, and skeletal homeostasis. **Table 1** provides a summary of recent animal studies investigating or linking the effects of GM on bone metabolism.

### Clinical observations in human studies

Clinical studies in human populations have provided further evidence supporting the association between GM dysbiosis and OP prevalence, BMD changes, and fracture risk. Analysis of GM composition in osteoporotic individuals has revealed alterations in GM diversity and abundance compared to healthy controls. Epidemiological studies have identified associations between dietary patterns, GM composition, and bone health outcomes. High-fiber diets rich in fruits, vegetables, and whole grains, which promote a diverse GM profile, have been associated with higher BMD and reduced fracture risk. Conversely, diets high in saturated fats, refined sugars, and processed foods, which disrupt GM composition, may contribute to bone loss and OP development. **Table 2** summarizes clinical studies examining GM alterations or interventions among patients with low BMD.

Overall, both experimental evidence from animal studies and clinical observations in human populations support the notion of a bidirectional interaction between GM and bone health. Further research is needed to elucidate the underlying mechanisms and determine the clinical utility of GM-targeted interventions for OP prevention and management.

## Therapeutic implications and management strategies

### GM-targeted interventions

GM-targeted interventions aim to modulate GM composition and activity to promote bone health and mitigate OP risk. Several approaches have been proposed and investigated in preclinical and clinical studies.

#### Probiotics

Probiotics are live microorganisms that confer health benefits when administered in adequate amounts (Nagpal et al., 2012). Certain probiotic strains, such as Lactobacillus and Bifidobacterium species, have been shown to positively influence bone metabolism and improve skeletal phenotypes in animal models (Schepper et al., 2017). Clinical trials investigating the efficacy of probiotic supplementation in improving BMD and reducing fracture risk in humans are ongoing.

#### Prebiotics

Prebiotics are non-digestible dietary fibers that selectively promote the growth and activity of beneficial gut bacteria (Davani-Davari et al., 2019). By fermenting prebiotic fibers, GM produce SCFAs and other metabolites with potential bone-protective effects. Dietary supplementation with prebiotics, such as inulin, oligofructose, and resistant starch, may enhance GM diversity and function, thereby improving bone health.

#### Synbiotics

Synbiotics are combinations of probiotics and prebiotics designed to synergistically promote GM balance and function (Roy and Dhaneshwar, 2023). By providing both beneficial microbes and substrates for their growth, synbiotics aim to optimize GM composition and activity. Clinical trials investigating the effects of synbiotic supplementation on bone health outcomes are underway.

#### Fecal microbiota transplantation

FMT is a medical procedure in which fecal matter from a healthy donor is transplanted into the gastrointestinal tract of a recipient. This procedure aims to restore a balanced GM in individuals suffering from conditions linked to dysbiosis, such as *Clostridioides difficile* infection and certain inflammatory bowel diseases (IBDs). While primarily used to treat gastrointestinal disorders, FMT may also impact systemic health outcomes, including bone metabolism (Biazzo and Deidda, 2022; Zhang et al., 2022c; Zheng et al., 2022).

Overall, targeting GM composition and activity through these interventions holds promise for optimizing bone metabolism, preserving BMD, and reducing fracture risk in individuals at risk of OP. However, further research is needed to elucidate the optimal dosing, duration, and efficacy of GM-targeted interventions for OP prevention and management.

### Dietary approaches and nutritional interventions for OP management

#### Calcium and vitamin D supplementation

Adequate intake of calcium and vitamin D is essential for bone mineralization and remodeling. Calcium-rich foods, such as dairy products, leafy greens, and fortified foods, should be consumed as part of a balanced diet. Vitamin D sources include fatty fish, fortified foods, and sunlight exposure. Supplementation may be necessary for individuals with inadequate dietary intake or limited sun exposure.

#### Protein intake

Protein is essential for bone formation and maintenance, as it provides amino acids necessary for collagen synthesis and bone matrix deposition (Devignes et al., 2022; Selvaraj et al., 2024). Consuming adequate protein from sources such as lean meats, poultry, fish, eggs, legumes, and dairy products supports bone health. However, excessive protein intake, particularly from animal sources, may have adverse effects on bone health and should be moderated.

#### Nutrient-rich diet

Consuming a nutrient-rich diet rich in fruits, vegetables, whole grains, and lean proteins provides essential vitamins, minerals, and antioxidants necessary for bone health. Phytochemicals found in plant-based foods may have beneficial effects on bone metabolism and reduce inflammation associated with OP.

#### Limiting sodium and caffeine

High sodium intake and excessive caffeine consumption have been associated with calcium excretion and bone loss (Heaney, 2002; Park et al., 2014; Reuter et al., 2021). Limiting sodium intake and moderating caffeine consumption from sources such as coffee, tea, and soda may help preserve BMD and reduce OP risk.

#### Alcohol moderation

Excessive alcohol consumption has been linked to decreased BMD and increased fracture risk. Moderating alcohol intake and avoiding binge drinking are recommended to support bone health.

#### Weight-bearing exercise

Engaging in weight-bearing and resistance exercises, such as walking, jogging, strength training, and yoga, helps stimulate bone formation and improve BMD. Regular physical activity is essential for maintaining bone strength and reducing OP risk.

By adopting a balanced diet rich in essential nutrients, limiting harmful dietary factors, and engaging in weight-bearing exercise, individuals can support bone health and mitigate the risk of OP. Dietary approaches and nutritional interventions complement GM-targeted interventions in promoting overall skeletal health and reducing fracture risk in susceptible populations.

## Future directions and challenges

As research on the GM-bone axis continues to evolve, several areas for future investigation and challenges in translating research findings into clinical practice warrant attention. Here, we discuss potential avenues for future research and the obstacles faced in applying research findings to clinical management.

### Areas for future research

In future research, elucidating mechanistic insights into the interaction between the GM and bone health is crucial. Investigating specific microbial species, metabolites, and signaling pathways can enhance understanding of the GM-bone axis, paving the way for novel therapeutic targets for OP. Additionally, longitudinal studies tracking changes in GM over time and their associations with bone outcomes are essential for establishing causal relationships and understanding temporal dynamics. Well-designed clinical trials are needed to evaluate the efficacy, safety, and long-term effects of GM-targeted interventions, such as probiotics and prebiotics, in improving bone health outcomes. Personalized medicine approaches, incorporating individual variability in GM composition and lifestyle factors, hold promise for tailoring OP prevention and treatment strategies to specific patient populations. Exploring the gut-brain-bone axis represents a promising area of research for uncovering novel pathways regulating bone metabolism and remodeling.

### Challenges in translating research findings into clinical practice

Translating research findings into clinical practice faces several challenges. Standardization of GM analysis methods and data reporting guidelines is necessary to ensure reproducibility and reliability of research in clinical settings. The heterogeneity of OP phenotypes, influenced by genetics, lifestyle factors, and comorbidities, complicates the identification of consistent biomarkers and therapeutic targets. Regulatory approval of GM-targeted therapies requires robust clinical evidence and careful consideration of safety concerns. One of the significant challenges in implementing probiotic and prebiotic therapies is ensuring patient adherence. Factors such as taste preferences, dietary habits, and the need for consistent long-term use can affect compliance. Improving adherence requires patient education on the benefits of these interventions, developing more palatable and convenient formulations, and integrating these therapies into daily routines in a manner that is easy to follow. Additionally, while short-term studies have demonstrated the benefits of probiotics, prebiotics, and FMT, long-term safety remains a concern. Potential risks include alterations in GM composition that could lead to negative health outcomes, interactions with existing medications, and the possibility of infections, especially with FMT. Therefore, more extensive long-term studies are needed to fully understand the safety profiles of these interventions. Furthermore, the regulatory environment for GM-based therapies is still developing. Probiotics and prebiotics are often classified as dietary supplements, which are subject to less stringent regulatory scrutiny compared to pharmaceuticals. FMT, due to its complexity and potential risks, involves more rigorous regulations. Navigating these regulatory requirements and obtaining necessary approvals can be significant hurdles. Collaboration between researchers, healthcare providers, and regulatory bodies is essential to establish clear guidelines and protocols for the safe and effective use of these therapies. Addressing these challenges and advancing research in the field of GM and OP will pave the way for personalized, evidence-based approaches to bone health management and fracture prevention in diverse patient populations. Collaborative efforts among researchers, clinicians, industry partners, and regulatory agencies are essential for realizing the potential of GM-targeted interventions in improving skeletal health and reducing the burden of OP on a global scale.

## Conclusion

The burgeoning field of research on the GM-bone axis has shed light on the intricate interplay between gut microbial communities and skeletal health. Through a comprehensive review of experimental evidence and clinical observations, this review has elucidated the multifaceted impact of GM on OP pathogenesis and management. In conclusion, unraveling the impact of GM on OP pathogenesis offers promising opportunities for personalized, evidence-based strategies to optimize bone health and reduce fracture risk. Collaborative endeavors across disciplines are essential for advancing our understanding of the GM-bone axis and translating scientific discoveries into tangible clinical benefits for individuals at risk of OP.

Moving forward, future research efforts should focus on elucidating mechanistic insights into the GM-bone axis, conducting longitudinal studies to establish causal relationships, and evaluating the efficacy of GM-targeted interventions in clinical trials. Challenges such as standardizing GM analysis, addressing heterogeneity in OP phenotypes, and translating research findings into clinical practice must be overcome to realize the potential of GM-based approaches in OP management.
